# Assessment of access and utilization of adolescent and youth sexual and reproductive health services in western Ethiopia

**DOI:** 10.1186/s12978-021-01136-5

**Published:** 2021-04-23

**Authors:** Temesgen Tilahun, Tariku Tesfaye Bekuma, Motuma Getachew, Assefa Seme

**Affiliations:** 1 School of Medicine, Institute of Health Sciences, Wollega University, Nekemte, Ethiopia; 2 Department of Public Health, Institute of Health Sciences, Wollega University, Nekemte, Ethiopia; 3 Department of Reproductive Health and Health Service Management, College of Health Sciences, Addis Ababa University, Addis Ababa, Ethiopia

**Keywords:** Adolescents, Youth, Sexual and Reproductive Health Services, East Wollega

## Abstract

**Background:**

Despite Ethiopia's enormous effort in youth-friendly service provision, little was investigated about the challenges of accessing sexual and reproductive health services in Western Ethiopia. Thus, this study aimed to assess factors associated with the utilization of adolescent and youth sexual and reproductive health services in this area.

**Methods:**

A community-based cross-sectional quantitative method mixed with the qualitative inquiry was conducted among 771 adolescents and youth aged 15 to 24 years from February 1 to 28, 2020. Data were collected through face-to-face interviews using pretested structured questionaries. Data were entered using EPI-INFO version 7.0 and analyzed by SPSS version 25. Descriptive analysis and logistic regressions were performed. The adjusted odds ratio with a 95% confidence interval was used and statistical significance was declared at P-value < 0.05. The qualitative inquiry was collected through in-depth interviews with service providers, focus group discussions, and observation checklists of service units in the study facilities. Data were analyzed thematically.

**Results:**

The mean age of participants was 18.99 years (SD ± 2.49). Two hundred seventeen (28.1%) of participants reported that they have ever heard about adolescents' and youth’s reproductive health services. Only 66 (8.6%) have ever visited health facilities for sexual and reproductive health (SRH) services. Factors associated with the utilization of sexual and reproductive health service were age from 15 to 19 years (AOR = 0.36; 95%CI: 0.17, 0.76), history of having sexual intercourse(AOR = 5.34;95%CI: 2.53, 11.23), ever heard about sexual reproductive health service (AOR = 11.33; 95%CI: 5.59, 22.96), and visited a health facility for other health services (AOR = 5.12; 95%CI:1.72,15.24).

**Conclusion:**

Sexual and reproductive health service utilization among adolescents and youth was found to be low. The factors associated with adolescents and youth sexual and reproductive health services utilization were age, history of ever having sexual intercourse, ever heard about SRH services, and visit the health facility for other services. Therefore, it is better if the concerned bodies work on improving awareness of adolescents and youth towards SRH services and integrating these services into other routine services.

## Plain Language Summary

**Background:**

Ethiopia is showing its enormous effort to improve the utilization of sexual and reproductive health services by adolescents and youths. But different factors are affecting it. This paper explains how many adolescents and youths are using sexual and reproductive health services and the factors affecting their utilization.

**Methods:**

We interviewed 771 adolescents and youths aged 15 to 24 years in western Ethiopia. We asked them about the awareness and utilization of adolescents’ and youths’ sexual reproductive health services and the factors affecting their utilization.

**Results:**

seven hundred seventy-one adolescents and youths were interviewed. Two hundred seventeen (28.1%) and 66 (8.6%) of them heard about and utilized adolescents' and youths' reproductive health services respectively. Those adolescents and youths who heard about sexual and reproductive health service practiced sexual intercourse and visited a health facility for other health services used sexual and reproductive health service more frequently.

**Conclusion:**

Adolescents and youths who were using Sexual and reproductive health services were not many. Therefore, it better if the concerned bodies work on improving awareness and utilization of adolescents' and youths' sexual and reproductive health services.

## Introduction

The world today is home to the largest generation of young people in history, 1.8 billion. Close to 90 percent of them live in developing countries [1]. The World Health Organization (WHO) defines adolescents as those between 10 and19 years of age & youth is a people between15 up to 24 years of age. [2]. Adolescence is a period of transition between childhood & adulthood. During this period, secondary sexual characteristics develop and other biological changes occur, as a result, adolescents experience sexual urges, a sexual exploration that leads them to exercise sexual practices which may be safe or unsafe. This is the time where most adolescents start experiencing sexual activities [3–5].

The concern about adolescent sexual and reproductive health (ASRH) had grown following reports that sexual activity, early pregnancies, and sexually transmitted infections (STIs) including human immune deficiency virus (HIV) infection rates were increasing at unprecedented rates among adolescents [6, 7]. Youths are usually mistakenly perceived as healthy and as if they do not need special health services [8, 9].

The 1994 International Conference on Population Development (ICPD) in Cairo, Egypt, recognized adolescent-friendly reproductive health services (AFRHS) as an appropriate and effective strategy to address the sexual and reproductive health (SRH) needs of adolescents [10]. Ensuring universal access to quality services, free of discrimination, coercion, or violence, has been a core aim of the SRHR community since 1994 and is seen as an essential aspect of reproductive rights [11].

Youth-friendly service delivery is about providing services based on a comprehensive understanding of what young people in that particular society or community want, rather than being based only on what providers believe they need. It is also based on an understanding of, and respect for, the realities of young people’s diverse sexual and reproductive lives. A necessary part of youth-friendly service provision, therefore, is awareness among the providers of the special difficulties that young people face in accessing sexual and reproductive health services. For example, inconvenient hours, legal and policy hurdles, concerns about confidentiality, fear of discrimination (in particular among sexually active girls), being treated with disrespect and high costs are among the factors that can inhibit young people’s ability to access services [12–14].

There are barriers that adolescents and youths face in obtaining the health services they need. These factors are related to the client, provider, and health delivery system [5, 15–22].

Ethiopia is striving to meet the SRH needs and rights of the country’s largely underserved adolescent and youth population. Despite the interest in youth-friendly service provision, little researches investigated universal access to sexual and reproductive services among local youths and the attitude of providers across local facilities. Therefore, this study aimed to assess factors associated with the utilization of adolescent and youth sexual and reproductive health services in this area.

## Methods

### Study area and setting

The study was conducted in the East Wollega zone of Oromia regional state, Western Ethiopia.

East Wollega is found in the west part of the Oromia regional state. Administratively, it is organized into 18 Woredas, 43 towns, and 287 rural kebeles. According to the Central Statistic Agency 2007 report, the total population was estimated to be 1.5 million. Nekemte, which is 331 km far from the capital city of the country, Addis Ababa, is the capital city of East Wollega. The study was conducted from February 1 to 28, 2020.

### Study Design

A Cross-sectional quantitative method mixed with the qualitative inquiry was employed.

### Source population

Adolescents and Youth aged 15 to 24 years who were living in the East Wollega zone.

### Study population

Adolescents and youths living in the selected districts during the study period. Secondary respondents were service providers.

### Sample size determination

Single population proportion formula was used to calculate the sample size with the following assumption. The proportion of adolescents who utilized adolescent and youth-friendly services which were 63.8% was taken [23]. The marginal error of 5%, design effect 2, and 95% confidence level. After adding a 10% non-response rate, the final sample size was 781.

Four focus group discussions (FGDs) and five in-depth interviews were undertaken considering the saturation of the information. The qualitative data were collected allowing for the incoming data to adequately answer the research questions and the specific study objectives. Adolescent and youth sexual and reproductive health service providers and facilities’ heads were purposively selected for in-depth interviews. FGD participants were purposively selected adolescents and youths who were exposed & never exposed to the AYSRH service. A checklist was also used among seven health facilities that deliver the service to check if they were providing the services as per the standard.

### Sampling procedures

A multistage sampling technique was used in this research. We listed all the districts in East Wollega. Then, seven districts were selected by lottery method. After that, the districts were further stratified by rural & urban kebeles to ensure representativeness of the study population. Kebeles were sub-divided by predetermined zones from which one zone was randomly selected. Then, a list of households (HHs) in the selected zones was obtained from the kebele. Health extension workers of the respective kebeles were contacted to identify the HHs where the target age groups were found. Finally, the required number of participants were interviewed from randomly selected households.

For the qualitative data collection, those health centers serving the study kebeles were considered. Observation of purposely selected health facilities and in-depth interviews with direct service providers were conducted. In the next step, local youths were purposively selected for focus group discussions.

### Study variables

#### Data collection procedure

Data were collected by 16 data collectors who know Afan Oromo and local adolescents’ and youths’ contexts. The quantitative data were collected through interviewer-administered interviews using adopted questionnaires from reviewed literature on adolescent and youth sexual and reproductive health services. Research assistants for the qualitative inquiry were recruited based on their experience in the qualitative study and knowledge of the research topic. With consent from the participants, all responses in in-depth interviews and focus group discussions were tape-recorded and notes were also taken during the interview. All the interviews were moderated by trained research assistants. Open-ended semi-structured questions were used to initiate the interview and discussions.

#### Data processing and analysis

The data were checked, entered, and cleaned using EPI-INFO version 7.0 and then exported to Statistical Package for Social Sciences (SPSS) software package version 23 for analysis. Using the odds ratio (OR) with a 95% limit of the confidence interval, the association of dependent and independent variables was identified, and their degree of association was computed. Potential confounding variables were controlled by using multiple logistic regression. Frequency count, percentages, and OR interpretation were done during the result write-up and data presentation.

The transcripts and English-translated in-depth interviews were analyzed using qualitative data analysis. Additionally, in-depth interviews with participants’ views that illustrate key concepts were used directly during analysis. Finally, qualitative findings were triangulated with the quantitative findings.

#### Data quality assurance

The training was given to the research assistants on the objective of the study, how to conduct interviews, and focus group discussions. Then the questionnaire, and FGDs, and in-depth interviews’ topic guides were pre-tested and necessary amendments were made based on experience from the field pretest. The data collection was daily monitored for completeness. Research assistants were monitoring the interviews to ensure that it focuses on the topic and objective of the study. Qualitative interviews were undertaken in Afan Oromo which was translated to English daily after the transcript was written.

## Results

### Sociodemographic characteristics of study participants

In this study, 771 adolescents and youths were participated making a response rate of 98.7%. The majority, 468 (60.7%), of the participants were males. The mean age of participants was 18.99 (SD ± 2.49) with more than half 456 (59.1%) belonging to the age group of 15–19 years. Three hundred eighty-six (50.1%) of the participants were rural residents. Almost all of the participants were from the Oromo ethnic group 750(97.3%) and were single 758(98.3%). About seven in ten 542(70.3%) of participants reported that they were in school. The majority, 413 (53.6%), of participants attended grade 9–12. Only 7 (0.9%) of participants never attended school (Table 1).

**Table 1:** Socio-demographic characteristics of study participants, East Wollega, 2020

Characteristics		Frequency	Percentage
Sex	Male	468	60.7
	Female	303	39.3
Age	15–19	456	59.1
	20–24	315	40.9
Residence	Urban	385	49.9
	Rural	386	50.1
Religion	Protestant	531	68.9
	Orthodox	206	26.7
	Muslim	33	4.3
	Others	1	0.1
Marital status	None married	758	98.3
	Widowed	5	0.6
	Separated	8	1
Ethnicity	Oromo	750	97.3
	Amhara	19	2.5
	Tigre	1	0.1
	Others	1	0.1
Youth education	Out of school	124	16.1
	In school	542	70.3
	Completed	105	13.6
Highest education attained	Never attended	7	0.9
	Grade 1–4	39	5.1
	Grade 5–8	184	23.9
	Grade 9–12	413	53.6
	College and above	128	16.6
Father’s education	Cannot read and write	250	32.4
	Read and write	208	27
	Grade 1–4	54	7
	Grade 5–8	78	10.1
	Grade 9–12	82	10.6
	College and above	99	12.8
Mother's education	Cannot read and write	428	55.5
	Read and write	171	22.2
	Grade 1–4	37	4.8
	Grade 5–8	68	8.8
	Grade 9–12	36	4.7
	College and above	31	4
Youth employment	Government employee	12	1.6
	Nongovernmental organization employee	2	0.3
	Not employed/no job	125	16.2
	Private job	127	16.5
	Student	505	65.5
Father’s occupation	Farmer	505	65.5
	Government employee	123	16
	Merchant	82	10.6
	Bartender/waiter	6	0.8
	Day labor	40	5.2
	Others	15	1.9
Mother's occupation	Housewife	576	74.7
	Government employee	42	5.4
	Merchant	64	8.3
	Private job	2	0.3
	Student	3	0.4
	Local drink seller	8	1.0
	Daily laborer	14	1.8
	House servant	61	7.9
	Others	1	0.1
With whom they live	Both parents	535	69.4
	Father only	12	1.6
	Mother only	57	7.4
	Spouse	42	5.4
	Relatives	55	7.1
	Friends/peers	29	3.8
	Others	41	5.3

### Reproductive characteristics of study participants

Out of the total adolescents and youths interviewed, 494 (64.1%) had a boy or girlfriend. Two hundred nineteen (28.4%) of participants had a history of sexual intercourse, out of which 174 (79.5%) had practiced sexual intercourse in the last 12 months. The mean age at first sex was 18.06 (SD ± 2.031) years. It was found that 18 (5.9%) female participants from the total interviewed females had a history of abortion. Concerning the history of health facility visits, of the total interviewed youths, 465 (60.31%) had ever visited a health facility for any type of services.

### Awareness & utilization of Adolescents & Youths Sexual Reproductive Health (AYSRH)services

**Fig. 1:**
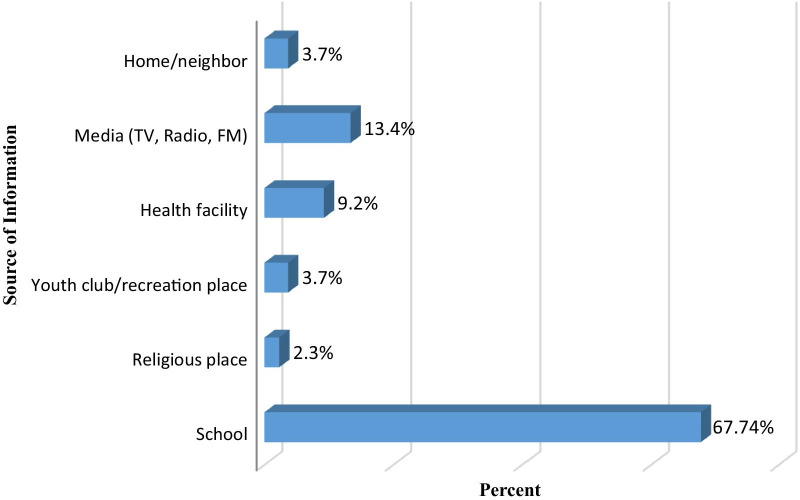
Source of Information about AYSRH services

Out of the total participants who had visited health facilities for any type of service, 50(10.32%) had ever seen adolescent and youth SRH services. From the total youths interviewed, more than one-fourth, 217 (28.1%) had heard about AYSRH services. Schools were the major source of information, 47 (67.74%) (Fig. 1). Among all participants who heard about AYSRH services, 99.08% and 79.27% of them got information about counseling and testing for HIV/AIDS, and contraception respectively.

**Fig. 2:**
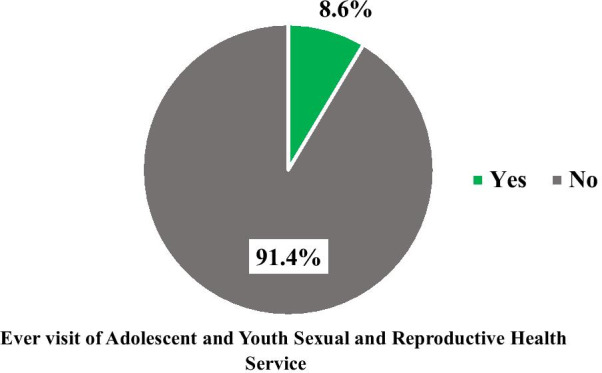
Proportion of adolescents and youths who ever visited Health facilities for AYSRH Services

Of the total participants, 66 (8.6%) had ever visited health facilities for adolescent and youth sexual and reproductive health services. This accounted for 30.41% of those who heard about AYSRH services (Fig. 2). Concerning the type of services ever used, the result showed that the majority, 43 (65.15%), of the participants had counseling and testing for HIV followed by contraception.

**Fig. 3:**
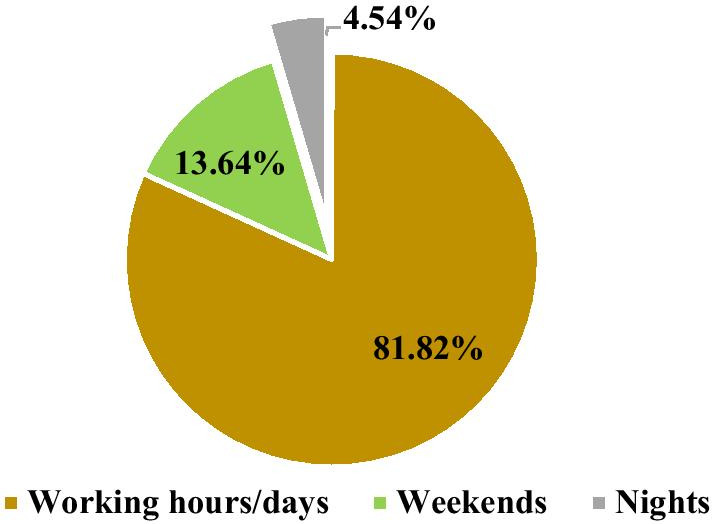
Time period of adolescents’ and youths’ most recent visit to a health facility for SRH services, East Wollega, 2020

The study also found that majority, 54 (81.82%) of the adolescents and youths who used the service had visited for the service during working hours (Fig. 3).

### Factors affecting adolescents and youths from accessing AYSRH services

In bivariate analysis, utilization of sexual and reproductive health services was significantly associated with variables like the educational status of participants, age of participants, having a boy or girlfriend, history of sexual practice, history of having a friend who has ever been pregnant, ever heard of adolescent and youths sexual and reproductive health services, ever visit the facilities for any other services and presence of parent-adolescent discussion about AYSRH services. After adjusting for confounding factors in the multivariate analysis, factors that were significantly associated with utilization of sexual and reproductive health service utilization were the age of participants, history of having sexual practice, ever heard of sexual and reproductive health services, and ever visited health facilities for any other types of services(Table 2).

**Table 2:** Multi-variate analysis results for factors affecting AYSRH service utilization, East Wollega, 2020

Independent variables		Ever used AYSRH services		Crude odds ratio (95% CI)	Adjusted odds ratio (95% CI)
		Yes	No		
Educational status of participant	Below high school	12	218	0.06	
	High school and above	54	487	2.01 (1.06, 3.84)	1.229 (0.48, 3.14)
Age group of participant	15–19 years	19	437	4.03 (2.32, 7.02)	0.36 (0.17, 0.76)*
	20–24 years	47	268	0.04	
Current school enrollment	Completed	14	91		
	In school	41	501	0.53 (0.28, 1.02)	1.99 (0.69, 5.75)
	Out of school	11	113	0.63 (0.27, 1.46)	0.68 (0.21, 2.22)
Participant’s mother’s educational level	No formal education	46	553	0.63 (0.36, 1.10)	0.89 (0.45, 1.79)
	Formal education	20	152	0.132	
Having boy/girl friend	Yes	57	437	3.88 (1.89, 7.97)	1.219 (0.51, 2.93)
	No	9	268	0.03	
Ever had sex	Yes	47	172	7.67 (4.38, 13.42)	5.33 (2.53, 11.23)*
	No	19	533	0.04	
Friend had been pregnant	Yes	26	108	0.24	
	No	40	597	0.28 (0.16, 0.48)	0.96 (0.49, 1.89)
Ever heard of AYSRH services	Yes	53	164	13.45 (7.15, 25.28)	11.33 (5.59, 22.96)*
	No	13	541	0.02	
Ever visited for any service	Yes	53	164	11.61 (4.18, 32.27)	5.11 (1.71, 15.23)*
	No	13	541	0.01	
Parent–adolescent discussion	Yes	20	100	2.63 (1.494, 4.63)	1.112 (0.55, 2.24)
	No	46	605	0.07	

^*^P-value < 0.05

Participants aged between 15 and 19 years were around 64% less likely to use AYSRH services compared to those aged between 20 and 24 years [AOR = 0.36;95%CI: 0.17, 0.76]. This was supported by the qualitative finding which explored that those who usually use the service were in their 20s to 30s most of whom were engaged.

Participants who had ever had sexual intercourse were more than 5 times likely to use the services compared to those who never started sexual intercourse [AOR = 5.33; 95%CI: 2.53, 11.23]. The qualitative findings also in line with this finding. For example, one key informant was asked about the most frequently used services in his facility and said, *“Both males and females always come after practicing unprotected sexual intercourse. Males come for condoms, and HIV testing and counseling while females come for pregnancy tests once they noticed missed menstrual cycle.”*

Participants who had ever heard about AYSRH services were more than 11 times likely to use the services compared to those who never heard about it [AOR = 11.33; 95%CI:5.59, 22.96]. Besides, participants who had ever visited health facilities for any health services had used AYSRH services 5 times more likely compared to participants who had never visited health facilities for any service [AOR = 5.12; 95%CI: 1.72, 15.24]. It was also explored in an in-depth interview that the youths who utilize the services are usually not exclusively coming for AYSRH service, rather they come for other services.

However, factors like participants' schooling status, family educational status, having a boy or girlfriend, and parent-adolescent discussion were not independently associated with utilization of AYSRH (Table 2).

## Discussion

This study had assessed access and utilization of adolescents' and youths' sexual and reproductive health services and related challenges. Information about sexual and reproductive health services is a precondition for adolescent and youth sexual and reproductive health (AYSRH) service utilization (17). In this study, 28.1% of participants were reported to have heard about adolescents' and youths' sexual and reproductive health (AYSRH) services. This finding is lower than studies conducted in Pakistan (52%) [19], Nigeria (82%) [20], Northwest Ethiopia (89.4%) [21], Bale Zone, Ethiopia (96.1%) [22],and Eastern Ethiopia (72.4%) [23]. This difference might be explained by, in this study, the inclusion of a greater number of participants from rural areas and out of schools where there is no access to information.

In this study, the majority of the participants reported that schools were the major source of information about AYSRH services. Similarly, a study conducted in Eastern Ethiopia among youths indicated that the main source of information on AYSRH was school teachers (31.5%) [23]. But, other studies conducted in other countries like Nigeria, Lagos (45.7%), and Pakistan, Labore (71%) revealed that the main source of information about adolescents and youths reproductive health services were friends [19, 20]. Mass media were the main source of information in Bale Zone, Ethiopia [22] and another study in Nigeria[24]. This difference might be a result of school-based AYSRH education campaign activities by health professionals in the current study areas.

In this study, 219 (28.4%) of the study participants ever had sexual intercourse. This is similar to a study conducted in Osun in Nigeria where 25.5% of adolescents had experienced sexual intercourse [24]. However, the finding in the current study is lower than that of study in Bale Zone, Ethiopia (39.5%) and North West Ethiopia (63.5%) [17, 22]. The observed difference might be attributed to the narrow geographical coverage in later studies than this study.

The utilization of AYSRH services was very low in the current study. Only 66 (8.6%) had ever visited health facilities for AYSRH services. This finding is lower than findings of similar studies conducted in Ethiopia among youths (63.8%) in Eastern Ethiopia [23], high school students in Bahirdar town (32%) [25]], and adolescents in Debre Berhan (33.8%) [26] and Northwest Ethiopia (18.4%) [25]. The finding is also lower than reports from studies conducted among youths aged between 15 and 24 years in Mynamar (67%) [27] and Makassar Indonesia (24.3%) [28]. The difference might be attributed to, in this study, the inclusion of a large number of participants from rural areas where access to information and health facility is limited.

In this study, the common AYSRH services adolescents and youths were using were HIV counseling & testing (65.15%) and contraceptives(40.91%). Similarly, more than half (60.57%) of youths in Northwest Ethiopia had used HIV counseling and testing [17]. Youths in Debre Berhan used HIV counseling and testing service(43.6%) followed by AYSRH information and education services (23.7%) [26]. A study in Myanmar among sub-urban youths aged between 15 and 24 years also showed family planning (70%) to be the most utilized service [27]. In the current study, utilization of other sexual and reproductive health services was low. Therefore, the focus should be given to improve access and utilization of AYSRH services in the study areas.

In this study, not all participants had received the services they need at the initial visit. It was found that 48 (72.73%) had received the services on the same day of their visit. However, a study conducted in Bale Zone, Ethiopia showed 95% of the youths who came to health facilities, received all services they need on the day of their visit [17]. This might be attributed to the differences in readiness of health facilities in terms of logistics, human power, and commitment of facility leaders. Study. The other possible explanation could be due to the work overload of care providers and the erratic supply of services in the current study as explained in the in-depth interview. For example, one male provider explained as “My work here is an additional job. I am primarily working in the expanded program for immunization (EPI) room where it has to be open always. When youths come for the service, I will ask them if their case is sensitive and will serve them at the EPI. Otherwise, I will either appoint on another day or refer them.”

This study showed that only 120(15.6%) of adolescents and youths reported that they had discussed at least one AYSRH topic with their family. This is lower compared to the studies conducted in Mecha District [17] and Awebal district (25.3%) [30] of Northwestern Ethiopia where respondents had a discussion on SRH issues with their families 32% and 25.5% respectively. The difference might be because the current study involved more participants from rural areas who are less encouraged to discuss SRH issues due to conservative cultural and religious practices than participants in later studies. Because societal norms like taboos of discussing sexuality with children are highly valued in rural communities.

Adolescents and youths Sexual and reproductive health services utilization were affected by different factors [17]. The current study identified factors like the previous history of sexual intercourse, age of participants, information about sexual and reproductive health services, and history of visiting health facilities for other services. Study participants who had ever had sexual intercourse were more than 5 times likely to use the services compared to those who never experienced sexual intercourse. This could be due to a difference in risk perception. Adolescents and youths who had sexual intercourse might relatively have a high level of risk perception for sexual and reproductive health-related than those who abstain. The same finding was also from similar studies conducted in Ethiopia [5, 17].

In this study, participants aged 15 to 19 years were 64% less likely to use adolescents and youths' sexual and reproductive health services compared to those aged from 20 to 24 years. This might be explained as those participants greater than 20 might be those who already engage in sexual intercourse as the median age at first sexual intercourse was found to be 18 years. This finding is consistent with a study conducted in Southern Ethiopia [29] and Northwest Ethiopia, Bahirdar [25] were those aged 20 to 24 used youth-friendly service more likely than those aged 15 to 19 years.

The participants who have ever had sexual intercourse practice were more than 5 times likely to use AYSRH services compared to those who did not practice it. This might be because those with experience of sexual intercourse might visit the health facilities either to get condoms, contraception, and HIV/AIDS counseling and testing or to get some interventions like abortion services when they experience negative consequences from unprotected sexual practice. This finding is in line with other similar studies conducted in Ethiopia [29, 30] where sexually active youths were more likely to use the youth-friendly services.

In this study, participants who have ever heard about SRH services had used the service more than 11 times likely compared to those who never heard about it. It is consistent with studies conducted in Mandalay city, Myanmar [27], in Indonesia, Makassar [28], in Nigeria, Lagos [20], and Eastern Ethiopia, Harar [23] which indicated that those who heard about the service were more likely to use the youth health service than those who never heard. This indicates raising awareness on SRH and the services improve utilization of AYSRH services.

Participants who have ever visited health facilities for any type of health services had used the SRH service 5 times more likely compared to the youths who have never visited facilities for any service. This might be due to those who came to health facilities for other services might hear the presence of AYSRH services from health professionals. Thus, integrating AYSRH services to other services in health facilities could improve its utilization.

## Conclusions

Sexual and reproductive health service utilization among adolescents and youths was found to be very low. The factors associated with adolescents' and youths' sexual and reproductive health services utilization were age, history of ever having sexual intercourse, ever heard about SRH services, and visit the health facility for other services. Therefore, it is better if the concerned bodies work on improving awareness of adolescents and youths towards SRH services and integrating these services into other routine services.

## Data Availability

The data sets used and analyzed during the current study are available from the corresponding author on reasonable request.

## Abbreviations

AIDS: Acquired immunodeficiency syndrome
AFRHS: Adolescent Friendly Reproductive Health Services
ART: Anti-retroviral therapy
AYFS: Adolescent and youth friendly services
CAC: Comprehensive abortion care
FGDs: Focus group discussions
HIV: Human immune virus
ICPD: International Conference for Population Development
IEC: Information Education and Communication materials
MSIE: Marie Stopes International Ethiopia
OR: Odds ratio
QDA: Qualitative data analysis
SPSS: Statistical package for the scientific solution
SRH: Sexual and reproductive health
STIs: Sexually transmitted infections
SRHR: Sexual and Reproductive Health Rights
SRHCS: Sexual and Reproductive Healthcare Services
TB: Tuberculosis
UNFPA: United Nations Fund for Population Activities
UNICEF: United Nations Children’s Fund
WHO: World Health Organization

## References

[1] Youth 2030 working with & young people: UN youth strategy. New York, 2018.

[2] UNAIDS. ‘UNAIDS terminology guidelines 2015’. www.unaids.org/en/resources/documents/2015/2015/terminologyguidelines

[3] Mulye TP, Park MJ, Nelson CD, Adams SH, Irwin CE, Brindis CD (2009). Trends in Adolescent and Young Adult Health in the United States. J Adolesc Health.

[4] World Health Organisation. Unsafe Abortion. Global and Regional Estimates of the Incidence of Abortion and Associated Mortality. 2008.

[5] Negash W (2016). Reproductive health service utilization and associated factors: the case of north Shewa zone youth, Amhara region Ethiopia. Pan Afr Med J.

[6] WHO, GTZ, GmbH. Sexually Transmitted Infections among Adolescents: the need for adequate health services. Geneva: 2005. 10.1016/s0968-8080(01)90021-7 11468834

[7] Malleshappa1 K, Shivaram K, Nandini C. Knowledge and attitude about reproductive health among rural adolescent girls in kuppam mandal an intervention study. Biomed Res. 2011; 22(3):305–10.

[8] Fonds C (2005). Training in Sexual Health Research.

[9] Bankole A, Malarcher S (2010). Removing barriers to adolescents’ access to contraceptive information and services. Stud Fam Plann.

[10] WHO. Programming for Adolescent Health and Development: Report of WHO/UNFPA/UNICEF Study Group on Programming for Adolescents Health. Geneva: WHO; 1999. 11400634

[11] OECD (2012). “Tackling the root causes of gender inequalities in the post-2015 development agenda”, submission to the global thematic consultation on addressing inequalities.

[12] Oronje RN, Crichton J, Theobald S, Lithur NO, Ibisom L (2011). Operationalising sexual and reproductive health and rights in sub-Saharan Africa: constraints, dilemmas and strategies. BMC Int Health Hum Rights.

[13] Chandra-Mouli V, Svanemyr J, Amin A, Fogstad H, Say L (2015). Twenty years after international conference on population and development: where are we with adolescent sexual and reproductive health and rights?. J Adolesc Health..

[14] UNFPA, 2016, Adolescent sexual and reproductive health, from www.unfpa.org/resources/adolescent-sexual-and-reproductive-health.

[15] WHO Making health services adolescent friendly: Developing national quality standards for adolescent friendly health Services: Geneva, 2012.

[16] Chandra-Mouli V, McCarraher DR, Phillips SJ, Williamson NE, Hainsworth G (2014). Contraception for adolescents in low and middle income countries: Needs, barriers, and access. Reproductive Health.

[17] Alehegn BG, Mulunesh TT, Yilkal TA, Abebaw AG (2018). Sexual and reproductive health services utilization and associated factors among preparatory school students in Mecha District, Northwest Ethiopia: cross sectional study. J Gynecol Women’s Health.

[18] Banerjee SK, Andersen KL, Warvadekar J, Aich P, Rawat A, Upadhyay B (2015). How prepared are young, rural women in India to address their sexual and reproductive health needs? a cross-sectional assessment of youth in Jharkhand. Reprod Health.

[19] Iqbal A (2017). Perceptions of adolescents’ sexual and reproductive health and rights: a cross-sectional study in Lahore District, Pakistan. BMC Int Health Human Rights..

[20] Ajike SO, Mbegbu VC (2016). Adolescent/youth utilization of reproductive health services: knowledge still a barrier. J Family Med Health Care.

[21] Khasay K (2016). Utilization of youth friendly services and associated factors in Mekelle town, Tigray Northern Ethiopia.. Int J Therap Appl..

[22] Kerbo AA, Tefera TB (2018). Youth Friendly Sexual and Reproductive Health Services Utilization and Associated Factors in Bale Zone of Ethiopia: A Community Based Cross Sectional Study. J Women’s Health Reprod Med..

[23] Motuma A, Syre T, Egata G, Kenay A (2016). Utilization of youth friendly services and associated factors among youth in Harar town, east Ethiopia: a mixed method study. BMC Health Serv Res.

[24] Omobuwa (2012). Knowledge and perception of reproductive health services among in-school adolescents in Ile-Ife, Osun State, Nigeria. J Med Med Sci.

[25] Abebe M, Awoke W (2014). Utilization of youth reproductive health services and associated factors among high school students in Bahir Dar, Amhara Regional State, Ethiopia. Open J Epidemiol.

[26] Tlaye (2018). Reproductive health services utilization and its associated factors among adolescent’s in Debre Berhan town Central Ethiopia: community-based cross-sectional study. Reproduct Health.

[27] Zaw T (2012). Equity of access to reproductive health services among youths in resource-limited suburban communities of Mandalay City Myanmar. BMC Health Serv Res.

[28] Violita and Hadi (2019). Determinants of adolescent reproductive health service utilization by senior high school students in Makassar Indonesia. BMC Public Health.

[29] Mulugeta B (2019). Assessment of youth-friendly service quality and associated factors at public health facilities in Southern Ethiopia: a facility-based cross-sectional study. Hindawi BioMed Res Int.

[30] A, Kassaw T, Hailu G, (2016). Level of young people sexual and reproductive health service utilization and its associated factors among young people in Awabel District Northwest Ethiopia. PLoS ONE.

